# Does the timed up and go test predict future falls among British community-dwelling older people? Prospective cohort study nested within a randomised controlled trial

**DOI:** 10.1186/s12877-015-0039-7

**Published:** 2015-04-03

**Authors:** Gotaro Kojima, Tahir Masud, Denise Kendrick, Richard Morris, Sheena Gawler, Jonathan Treml, Steve Iliffe

**Affiliations:** Department of Primary Care & Population Health, University College London, London, UK; Department of Health Care for Older People, Nottingham University Hospitals NHS Trust, Nottingham, UK; School of Medicine, Division of Primary Care, University of Nottingham, Nottingham, UK; University Hospitals Birmingham NHS Trust, Birmingham, UK

**Keywords:** Timed up and go test, Falls, Older people

## Abstract

**Background:**

Falling is common among older people. The Timed-Up-and-Go Test (TUG) is recommended as a screening tool for falls but its predictive value has been challenged. The objectives of this study were to examine the ability of TUG to predict future falls and to estimate the optimal cut-off point to identify those with higher risk for future falls.

**Methods:**

This is a prospective cohort study nested within a randomised controlled trial including 259 British community-dwelling older people ≥65 years undergoing usual care. TUG was measured at baseline. Prospective diaries captured falls over 24 weeks. A Receiver Operating Characteristic curve analysis determined the optimal cut-off point to classify future falls risk with sensitivity, specificity, and predictive values of TUG times. Logistic regression models examined future falls risk by TUG time.

**Results:**

Sixty participants (23%) fell during the 24 weeks. The area under the curve was 0.58 (95% confidence interval (95% CI) = 0.49-0.67, p = 0.06), suggesting limited predictive value. The optimal cut-off point was 12.6 seconds and the corresponding sensitivity, specificity, and positive and negative predictive values were 30.5%, 89.5%, 46.2%, and 81.4%. Logistic regression models showed each second increase in TUG time (adjusted for age, gender, comorbidities, medications and past history of two falls) was significantly associated with future falls (adjusted odds ratio (OR) = 1.09, 95% CI = 1.00-1.19, p = 0.05). A TUG time ≥12.6 seconds (adjusted OR = 3.94, 95% CI = 1.69-9.21, p = 0.002) was significantly associated with future falls, after the same adjustments.

**Conclusions:**

TUG times were significantly and independently associated with future falls. The ability of TUG to predict future falls was limited but with high specificity and negative predictive value. TUG may be most useful in ruling in those with a high risk of falling rather than as a primary measure in the ascertainment of risk.

## Background

More than one third of people aged 65 years or older fall every year and the prevalence of falling increases to 50% in those 80 years or older [1]. Falls are a leading cause of morbidity and mortality in older people and are associated with various negative health outcomes including fracture, functional decline, fear of falling, loss of confidence, and reduced quality of life [2-6]. In addition, healthcare costs attributable to falling are substantial and are expected to increase as the population ages [7]. Therefore, falling of older people has been recognised as a major but potentially preventable public health problem [2,3]. A number of interventions have been shown to decrease fall rates by 20-40% [8]. Accurate detection of those at high risk for falling and implementation of appropriate interventions could potentially avoid the negative impacts of falling.

Although multiple tools have been developed to identify older people at risk of falling [9], it is not known which tool has the best discriminative ability to predict falls risk. The Timed Up and Go test (TUG) is used frequently in both clinical and research settings [9,10]. This is a brief test not requiring special equipment and is suitable for use in primary care. TUG is one of a range of measures identified in clinical guidelines [4,11] as a possible screening tool to evaluate gait and balance functions and to identify older people at risk of falling.

While retrospective studies consistently demonstrated significant positive relationships between TUG time and history of falls, the predictive ability of TUG to identify future falls risk has recently been challenged by meta-analyses [12-14]. However, these meta-analyses included heterogeneous populations and some of the study cohorts were small. Only a limited number of studies validated TUG among large cohorts of community-dwelling older people [15-21]. Furthermore, these studies were from the US [15-17], Ireland [18], Norway [19], Taiwan [20], and Japan [21]; and no study has investigated TUG as a predictor of future falls among community-dwelling older people in England.

The main purposes of this study with British community-dwelling older people aged 65 years and older were: (1) to examine the ability of TUG (as either a continuous or a dichotomous variable) to predict future falls; 2) to determine the optimal cut-off point for TUG to correctly classify those with higher future falls risk.

## Methods

### Study population

This study used the data of British community-dwelling older people in the usual care arm of a randomised controlled trial, ProAct65 + [22,23]. This trial was a three-arm parallel design cluster randomised controlled trial conducted in London and Nottingham/Derby in 2008–2013 to examine the effects of two exercise programmes. The primary outcome was achievement of 150 minutes of moderate intensity physical activity per week twelve months after the interventions [22,23]. People aged 65 years and older who were able to walk independently and to participate in group exercise classes were recruited by participating general practices. Those who had three or more falls in the previous year or unstable medical conditions or were already exercising for 150 minutes/week or more were excluded. This trial was approved by Nottingham Research Ethics Committee 2, National Health Service Nottinghamshire County and Westminster, Brent, Harrow, Hounslow, and Barnet & Enfield Primary Care Trusts, and registered in ClinicalTrials.gov (NCT00726531) and ISRCTN (ISRCTN43453770). Written informed consent was obtained from all participants. A total of 1254 participants were enrolled in the trial and 457 were allocated to the usual care arm. Although participants in the usual care arm were not offered the trial exercise programmes, they were free to participate in any other exercise opportunities. Nineteen participants who did not undertake TUG tests, two participants who were found to have had three or more falls in the last year, and 177 participants who did not return more than half of falls diaries (see below) were excluded, leaving 259 participants (56.7%) as a final analytic sample for this study.

### Timed up and go test

At the baseline assessment, participants performed the TUG test, in which they were observed and timed as they stood up from a chair, walked three meters, turned around, walked back to the chair, and sat down [10]. A TUG time is the time in seconds that participants needed to complete the test. Longer time indicates worse balance and mobility performance.

### Prospective falls assessment

Falls were monitored prospectively over the 24-week study period using falls diaries. The falls diaries were mailed to each participant every four weeks, a total of six diaries [24]. All participants who did not return the diary were reminded by phone call. A fall was defined as an event of unintentionally coming to rest on the ground, floor, or other lower levels [1].

### Other measurements

Socio-demographic and clinical information collected at baseline included age, gender, height, weight, ethnicity, living situation (living alone or not), highest level of education achieved, annual household income, numbers of comorbidities and medications, and number of falls in the previous year. Body mass index (BMI) was calculated as weight in kilograms divided by square of height in meters.

### Data analyses

Participants who fell at least once during the 24-week follow-up period were defined as fallers and participants who did not fall were defined as non-fallers. Mean values of continuous variables in the baseline socio-demographic and clinical dataset were compared between fallers and non-fallers using independent t-tests. The chi-squared test was used to examine differences in proportion of categorical variables.

The predictive ability of TUG in identifying future falls was determined by using receiver operating characteristic (ROC) curve and area under the curve (AUC) analyses. Greater AUC indicates better predictive ability to identify future falls, ranging from 0.5, where the predictor is no better than chance, up to 1, indicative of 100% predictive ability.

The optimal cut-off point for TUG to correctly classify those with future falls risk from those without, and sensitivity, specificity, positive predictive value, and negative predictive value were calculated by ROC curve analysis. The time with highest Youden’s index (sensitivity + specificity - 1) [25] was determined to be the optimal cut-off point.

In logistic regression analysis, TUG time was used as a continuous variable as well as a dichotomous variable based on the cut-off point identified in the ROC curve analysis. First, unadjusted logistic regression models were used to calculate odds ratios (OR) with 95% confidence intervals (95% CI) of TUG time, TUG time above the cut-off point, and the other variables at baseline for prospective fall risk. Subsequently multivariable logistic regression models controlling for age, gender, and the variables significant in the unadjusted logistic regression models calculated OR with 95% CI for continuous and dichotomous TUG times for independent prospective fall risk.

A two-sided significance test was used and p value of <0.05 was considered statistically significant. All statistical analyses were performed with IBM SPSS Statistics (version 20, IBM Corporation, Armonk, NY, USA).

## Results

Of the entire cohort (N = 259), 59 participants (22.8%) had one or more falls over the 24-week follow-up period and were defined as fallers. Of those, one fall was reported by 38 participants (64.4%), two falls by 11 (18.6%), three falls by 6 (10.2%), four falls by 2 (3.4%), and both five and seven falls by one each (1.7% each). Baseline characteristics of the entire cohort, fallers, and non-fallers were described in Table 1. The entire cohort had a mean age of 72.6 years and 164 participants (63.3%) were women. More than 90% described their ethnic origin as White, and mean numbers of comorbidities and medications were approximately two and four, respectively. The fallers had significantly slower mean performance on TUG (11.4 vs. 10.1 seconds, p = 0.03) more comorbidities (2.6 vs. 1.9, p = 0.02) and medications (4.7 vs. 3.6, p = 0.03), and were more likely to have history of two falls in the previous year (13.6% vs. 2.0%, p < 0.001) compared with non-fallers. There were no significant differences between fallers and non-fallers in age, gender, BMI, ethnicity, living situation (living alone or not), education, income, enrollment site, number of falls in the previous year, and any falls in the previous year.

**Table 1:** **Baseline characteristics for entire cohort, fallers, and non-fallers**

Variable*	**Entire cohort**	**Fallers**	**Non-fallers**	**p value**
	**N = 259**	**n = 59**	**n = 200**	
Timed Up and Go test	10.4 ± 3.5	11.4 ± 4.2	10.1 ± 3.2	0.03
Age	72.6 ± 5.9	73.0 ± 6.2	72.6 ± 5.8	0.64
Age group				
65-69	94 (36.3%)	20 (33.9%)	74 (37.0%)	0.91
70-74	82 (31.7%)	20 (33.3%)	63 (31.5%)	
75-79	41 (15.8%)	11 (18.3%)	30 (15.0%)	
80+	42 (16.2%)	9 (15.3%)	33 (16.5%)	
Female	164 (63.3%)	40 (67.8%)	124 (62.0%)	0.42
Body mass index	26.7 ± 5.1	26.8 ± 3.6	26.7 ± 5.4	0.77
White ethnicity	233 (90.7%)	56 (94.9%)	177 (89.4%)	0.20
Living alone	80 (30.9%)	24 (40.7%)	56 (28.0%)	0.06
Education				
College/University	127 (49.2%)	29 (49.2%)	98 (49.2%)	0.99
Primary/Secondary	131 (50.8%)	30 (50.8%)	101 (51.8%)	
Income				
£20001+	88 (38.4%)	26 (48.1%)	62 (35.4%)	0.09
up to £20000	141 (61.6%)	28 (51.9%)	113 (64.6%)	
Site				
London	105 (40.5%)	23 (39.0%)	82 (41.0%)	0.78
Nottingham	154 (59.5%)	36 (61.0%)	118 (59.0%)	
Number of comorbidities	2.0 ± 1.7	2.6 ± 2.0	1.9 ± 1.5	0.02
Number of medications	3.9 ± 3.2	4.7 ± 3.8	3.6 ± 3.0	0.03
Number of falls in the previous year	0.3 ± 0.5	0.4 ± 0.7	0.3 ± 0.5	0.08
Any falls in the previous year	62 (23.9%)	17 (28.8%)	45 (22.5%)	0.32
Two falls in the previous year	12 (4.6%)	8 (13.6%)	4 (2.0%)	<0.001

*mean ± standard deviation or n (%).

**Figure 1:**
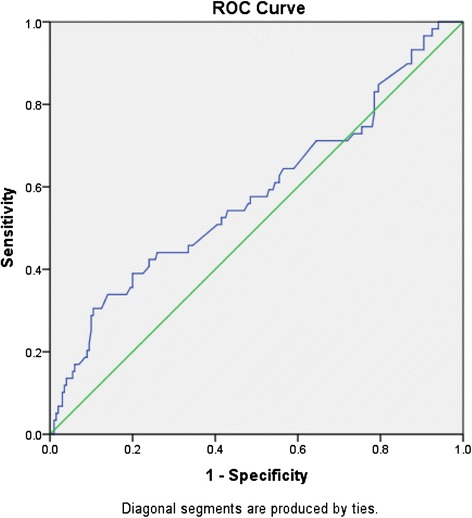
**Area under the curve = 0.58, 95% confidence interval = 0.49-0.67, p = 0.06.**

ROC curve of TUG times as a predictor of future falls showed the AUC was 0.58 (95% CI = 0.49-0.67, p = 0.06), which indicates limited predictive ability (Figure 1). The highest Youden’s index was 0.20 with a cut-off point of 12.6 seconds. With this cut-off point, TUG had 30.5% sensitivity, 89.5% specificity, 46.2% positive predictive value, and 81.4% negative predictive value. The positive and negative likelihood ratios were 2.91 and 0.78, respectively. TUG correctly predicted at baseline whether or not participants would fall over the 24 weeks in 197 out of 259 participants (accuracy = 76.1%).

Univariable logistic regression models demonstrated slower performance in TUG, higher numbers of comorbidities and medications, and two falls in the previous year at baseline were significantly associated with falling during the subsequent 24-week period (Table 2). Specifically, each one second increase in TUG time was associated with 10% increased odds for future falls and those with TUG times of ≥12.6 seconds were 3.7 times more likely to have future falls than those with TUG times of <12.6 seconds. Multivariable logistic regression models controlling for age, gender, numbers of comorbidities and medications, and two falls in the previous year were used to calculate ORs for the predictive value of TUG times (Table 3). Each one second increase in TUG time was significantly and independently associated with the odds of future falls (Model 1: OR = 1.09, 95% CI = 1.00-1.19, p = 0.05). A dichotomised TUG time with a cut point at ≥12.6 seconds was also associated with the odds of future falls, adjusted for age, gender, numbers of comorbidities and medications, and two falls in the previous year (Model 2: OR = 3.94, 95% CI = 1.69-9.21, p = 0.002).

**Table 2:** **Univariate logistic regression models predicting falls during the 24-week follow-up**

**Variable**	**Odds ratio**	**p value**
TUG (continuous)	1.10 (1.02-1.19)	0.02
TUG ≥ 12.6 seconds	3.74 (1.83-7.65)	<0.001
Age	1.01 (0.96-1.06)	0.63
Female gender	1.30 (0.70-2.39)	0.42
Body mass index	1.01 (0.95-1.07)	0.82
White ethnicity	2.22 (0.64-7.70)	0.21
Living alone	1.76 (0.96-3.23)	0.07
Higher education (college or university)	1.00 (0.56-1.78)	0.99
Higher income (≥£20001)	1.63 (0.91-3.14)	0.10
Living in London	0.92 (0.51-1.67)	0.78
Number of comorbidities	1.26 (1.07-1.49)	<0.01
Number of medications	1.10 (1.00-1.20)	0.03
Any falls in the previous year	1.39 (0.73-2.68)	0.32
Two falls in the previous year	7.69 (2.23-26.54)	0.001

**Table 3:** Multivariable logistic regression models predicting falls during the 24-week follow-up *

**Variable**	**Model 1**		**Model 2**	
	**Odds ratio**	**p value**	**Odds ratio**	**p value**
TUG (continuous)	1.09 (1.00-1.19)	0.05	-	-
TUG ≥ 12.6 seconds	-	-	3.94 (1.69-9.21)	<0.01
Age	0.99 (0.93-1.04)	0.60	0.97 (0.92-1.03)	0.29
Female gender	1.41 (0.72-2.74)	0.32	1.45 (0.74-2.86)	0.28
Number of comorbidities	1.15 (0.91-1.45)	0.25	1.19 (0.94-1.51)	0.15
Number of medications	1.04 (0.92-1.17)	0.58	1.01 (0.89-1.15)	0.84
Two falls in the previous year	7.41 (2.03-27.04)	<0.01	7.18 (1.92-26.90)	<0.01

Continuous TUG time was used in Model 1 and dichotomous TUG with the cut-off point of 12.6 seconds was used in Model 2, both controlling for age, gender, numbers of comorbidities and medications, and two falls in the previous year.

## Discussions

In this study of 259 community-dwelling older people in the UK, the optimal cut-off point of TUG time to predict future falling was 12.6 seconds, with low sensitivity (30.5%) and high specificity (89.5%). Each one second increase in TUG time was associated with 9% increased odds for future falls, and TUG time > =12.6 seconds was associated with approximately four times higher odds of future falls compared with TUG time < 12.6 seconds. However, ROC curve analysis showed that TUG had limited predictive ability for future falls.

Several prospective studies have examined TUG’s future fall predictive ability among community-dwelling older people [15-21]. In our ROC curve analysis, the AUC for TUG times to predict future falls was 0.58, which indicates limited predictive value. The AUC calculated by other studies ranged from 0.50 to 0.72 [15-18,20]. A consensus has not been reached regarding the general cut-off point for TUG to correctly classify those at high future falls risk. Two of the previous prospective studies demonstrated the optimal cut-off points of 15.3 and 16 seconds [18,21] and other small-sized studies (N = 60 and 35) showed 12.5 and 12.3 seconds [26,27], which are similar to our result of 12.6 seconds. With this cut-off point, we found TUG had low sensitivity but high specificity in identifying those who will fall.

The limited predictive ability for future falls may be attributed to the fact that the cause of falling in older people is multifactorial [2]. Although the TUG test is able to evaluate basic balance and mobility function, it may not be comprehensive enough to cover other falls risk components, such as environmental or intrinsic factors [2]. In general, falls risk screening tools, including TUG, have been shown to have limited to moderate ability to predict future falls with higher specificity than sensitivity [9,12-14], as in our study.

Logistic regression models were performed in a few prospective studies to examine association between TUG and future falls risk, showing each second increase in TUG time was associated with only 2-3% higher risk for future falls [20,21] while a 9% higher future falls risk was observed in our study. Our higher fall risk may be attributed to our rigorous falls monitoring system with daily falls diaries, which may have led to more accurate fall detection compared with phone call every 3 months plus postcard to be sent when falling [20] or a one-time retrospective self-report questionnaire [21].

There are some limitations in this study. Firstly, since our study sample was older people living in the community who had been recruited for an exercise intervention trial, participants may not be representative of general community-dwelling older population. They may also have been more motivated to undertake exercise and more aware of falls and fall risks than the general older people. Secondly, since participants were living in London or Nottingham/Derby, UK and more than 90% of them were White, our findings may not be generalizable to those in other geographic areas or with other ethnicity backgrounds. Lastly, capturing falls was based on self-report by participants, which may have affected our results.

A major strength of the current study is high quality falls incidence data. In order to detect falls, we used fall diaries to be recorded by participants daily and submitted every four weeks along with follow-up phone calls as necessary. This prospective robust monitoring system with the standardised tool should have minimised recall bias [28]. It was demonstrated in the same trial cohort that those with more fall risk factors were less likely to return the diaries but more likely to have falls [24]. Therefore we included only those who returned more than half of the fall diaries in the analyses to avoid underreporting fall incidence [24].

Another strength is the potential implications of our findings for clinical practice. In the UK and other countries, general practice is often the main source of referral to exercise programmes and to falls clinics [29,30]. Our sample of older people who were recruited in general practices and volunteered for the exercise intervention trial is likely to represent those who should be evaluated in general practice for fall risks and, if necessary, referred in line with national guidance. The optimal cut-off point in this study has low sensitivity, high specificity and high negative predictive value. It indicates that although TUG time may not be additive to falls history and simple gait observation [11] in identifying those requiring further diagnosis, assessment and intervention, it may help to exclude those at low risk.

## Conclusions

In conclusion, although the TUG test’s limited ability to predict future falls restricts its utility as a routine falls *screening* tool among British community-dwelling older people, the high specificity and negative predictive value at the 12.6 seconds threshold makes it useful *clinically* in those who have a high falls risk.

## Abbreviations

AUC: Area under the curve
OR: Odds ratio
ROC: Receiver operating characteristic
TUG: Timed up and go test
